# Supplementary material for: Protective effect of luteinizing hormone on frozen-thawed ovarian follicles and granulosa cells
Source: PLoS One. 2025 Jan 14;20(1):e0317416. doi: 10.1371/journal.pone.0317416 (PMC11731763; doi:10.1371/journal.pone.0317416)
Supplement: S1 File — (PDF) [file pone.0317416.s001.pdf]

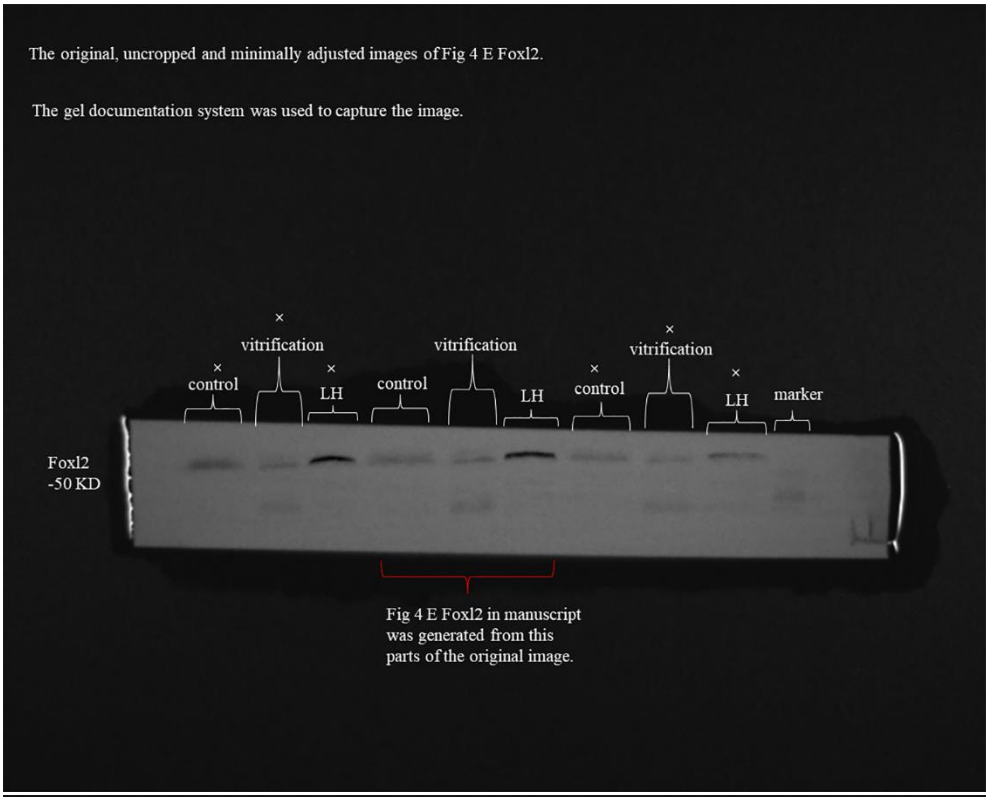
The original, uncropped and minimally adjusted images of Fig 4 E Foxl2.
The gel documentation system was used to capture the image.
×
vitrification
×
control
×
LH
control
vitrification
LH
×
control
×
vitrification
×
LH
marker
Foxl2
-50 KD
Fig 4 E Foxl2 in manuscript
was generated from this
parts of the original image.

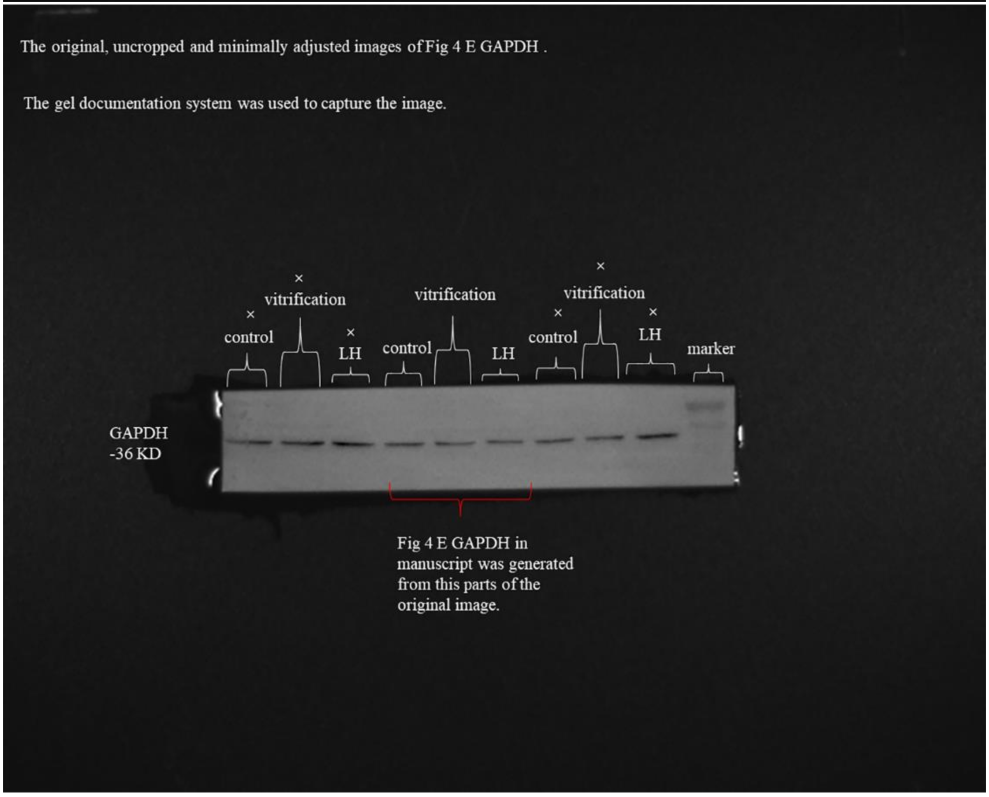
The original, uncropped and minimally adjusted images of Fig 4 E GAPDH .
The gel documentation system was used to capture the image.
×
vitrification
×
control
×
LH
control
vitrification
LH
×
control
×
vitrification
×
LH
marker
GAPDH
-36 KD
Fig 4 E GAPDH in
manuscript was generated
from this parts of the
original image.

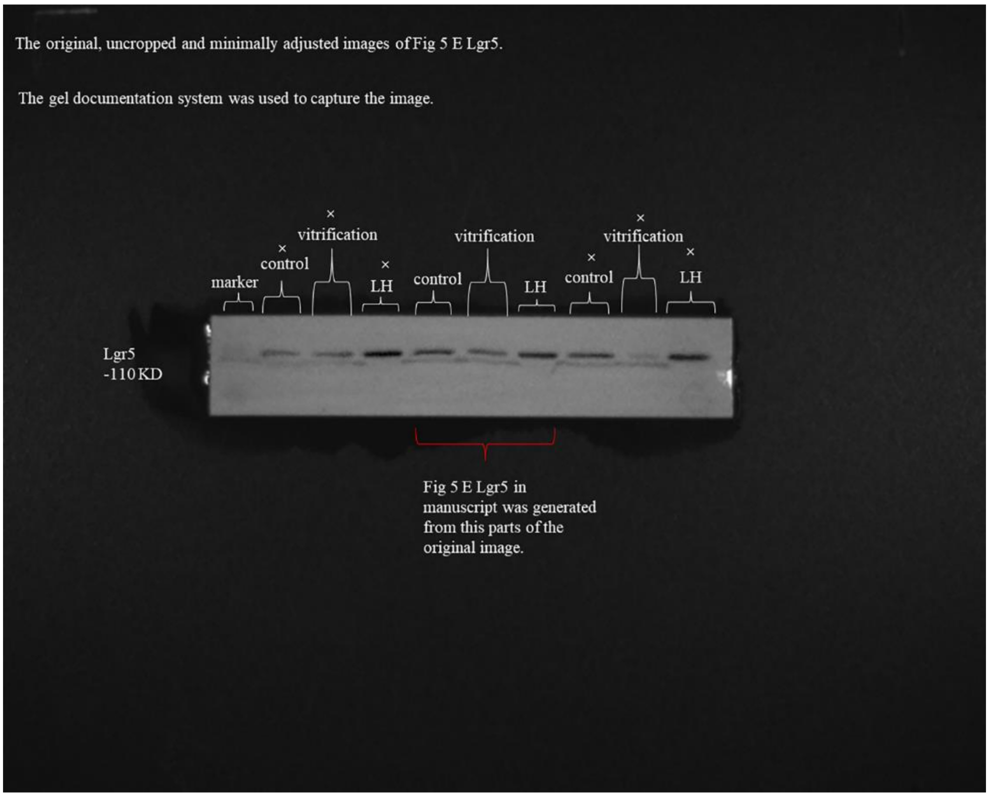
The original, uncropped and minimally adjusted images of Fig 5 E Lgr5.
The gel documentation system was used to capture the image.
×
vitrification
×
control
×
LH
marker
control
vitrification
LH
×
vitrification
×
control
×
LH
Lgr5
-110 KD
Fig 5 E Lgr5 in manuscript was generated from this parts of the original image.

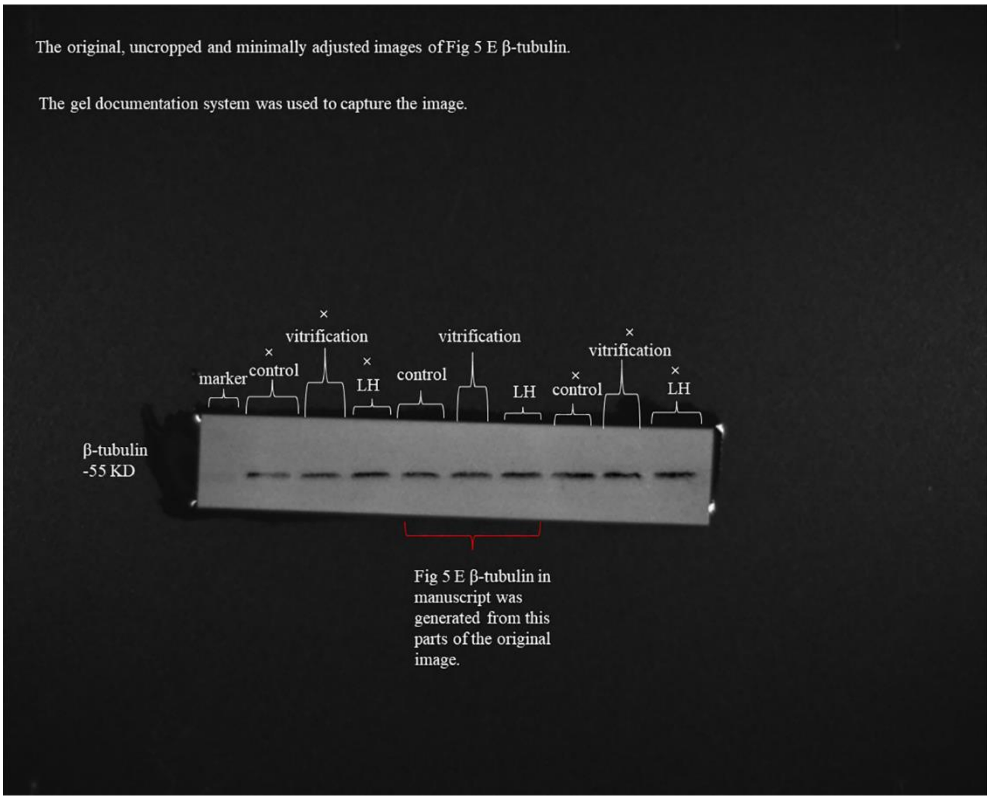
The original, uncropped and minimally adjusted images of Fig 5 E β-tubulin.
The gel documentation system was used to capture the image.
×
vitrification
×
control
×
LH
marker
control
vitrification
LH
×
vitrification
×
control
×
LH
β-tubulin
-55 KD
Fig 5 E β-tubulin in manuscript was generated from this parts of the original image.

The original, uncropped and minimally adjusted images of Fig 6 D LHR.

The eBLOT Touch Imager XLi was used to capture the image.

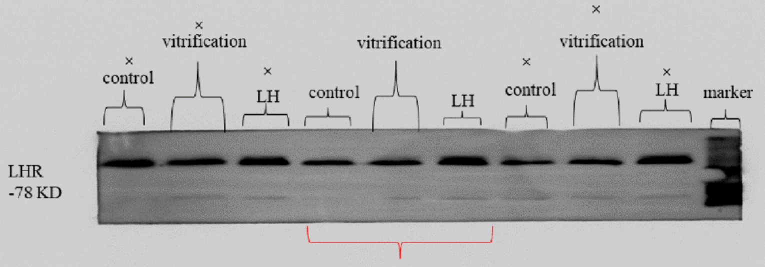

Fig 6 D LHR in manuscript was generated from this parts of the original image.

The original, uncropped and minimally adjusted images of Fig 6 D GAPDH.

The eBLOT Touch Imager XLi was used to capture the image.

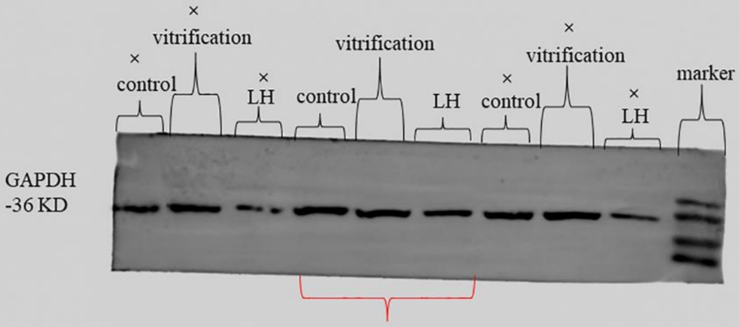

Fig 6 D GAPDH in manuscript was generated from this parts of the original image.

The original, uncropped and minimally adjusted images of Fig 7 D active caspase-3.

The eBLOT Touch Imager XLi was used to capture the image.

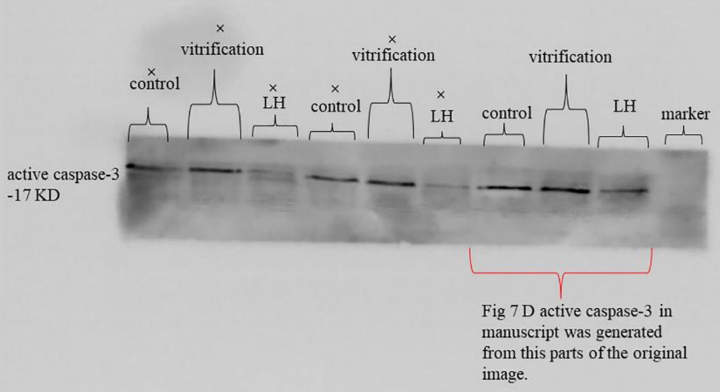

The original, uncropped and minimally adjusted images of Fig 7 D β-tubulin.

The eBLOT Touch Imager XLi was used to capture the image.

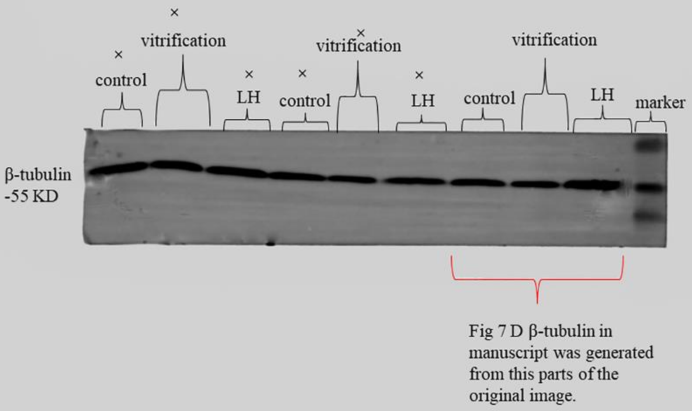

The original, uncropped and minimally adjusted images of Fig 8 E TGF-β.

The eBLOT Touch Imager XLi was used to capture the image.

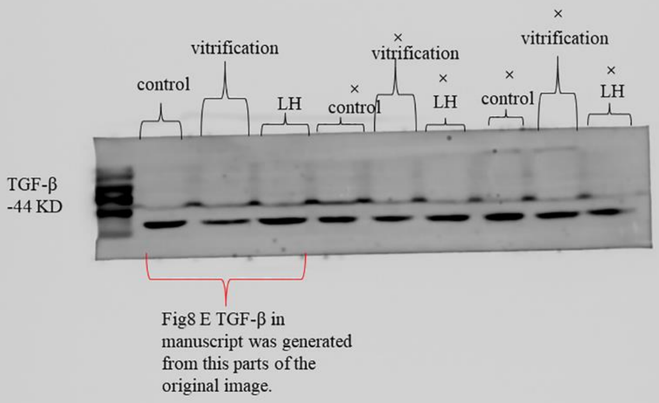

The original, uncropped and minimally adjusted images of Fig 8 E GAPDH.

The eBLOT Touch Imager XLi was used to capture the image.

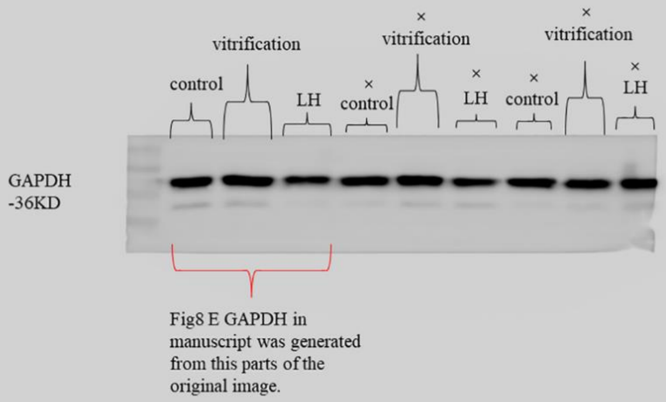

The original, uncropped and minimally adjusted images of Fig 8 F GDF-9.

The eBLOT Touch Imager XLi was used to capture the image.

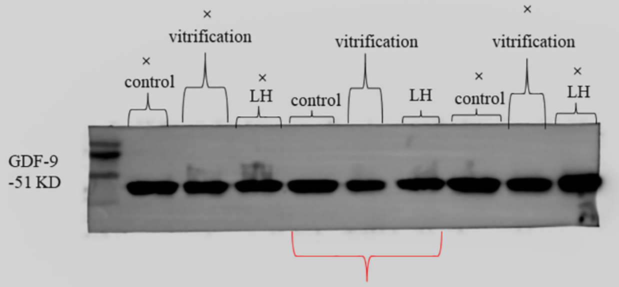

Fig8 F GDF-9 in manuscript was generated from this parts of the original image.

The original, uncropped and minimally adjusted images of Fig 8 F GAPDH.

The eBLOT Touch Imager XLi was used to capture the image.

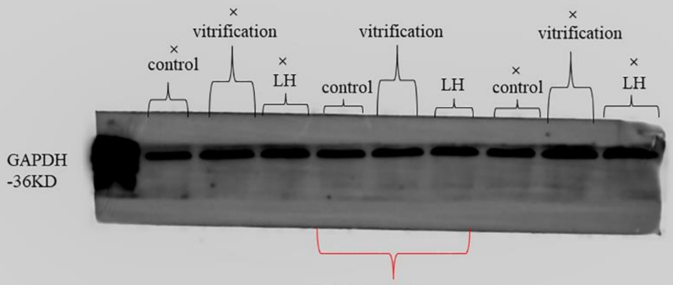

Fig8 F GAPDH in manuscript was generated from this parts of the original image.

The original, uncropped and minimally adjusted images of Fig 8 G BMP-15.

The eBLOT Touch Imager XLi was used to capture the image.

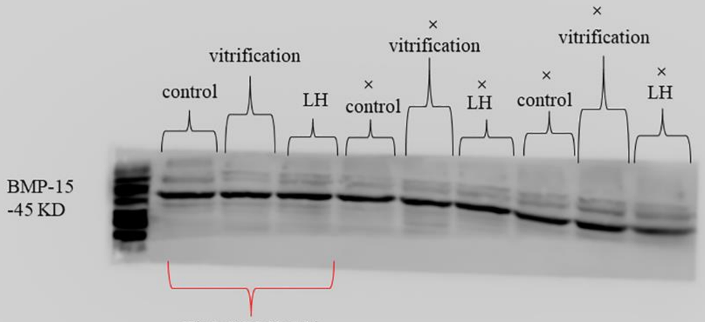

Fig8 G BMP-15 in manuscript was generated from this parts of the original image.

The original, uncropped and minimally adjusted images of Fig 8 G GAPDH.

The eBLOT Touch Imager XLi was used to capture the image.

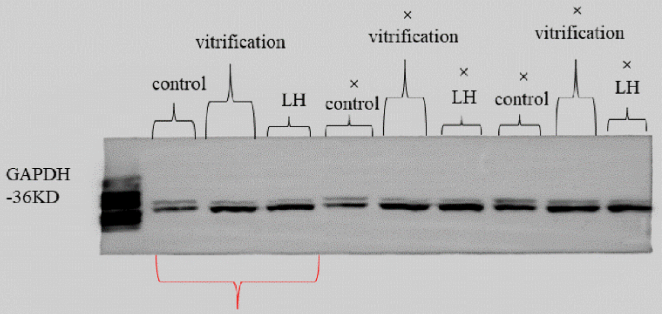

Fig8 G GAPDH in manuscript was generated from this parts of the original image.
