# Supplementary material for: Protective effect of luteinizing hormone on frozen-thawed ovarian follicles and granulosa cells
Source: PLoS One. 2025 Jan 14;20(1):e0317416. doi: 10.1371/journal.pone.0317416 (PMC11731763; doi:10.1371/journal.pone.0317416)

Raw data of Fig 2:

Fig2 C					
Number of repetitions	Cell count(per ml)	control	LH-BV	LH-AV	LH-TV
1	LIVE cell cout	7.19	4.02	3.51	7.21
2	LIVE cell cout	6.52	4.77	3.78	7.05
3	LIVE cell cout	6.11	4.69	4.21	8.12
1	Dead cell cout	2.81	5.98	6.49	2.79
2	Dead cell cout	3.48	5.23	6.22	2.95
3	Dead cell cout	3.89	5.31	5.79	1.88

Fig2 C-F(histogram data)							
Cell count(per ml)(mean)	control	Cell count(per ml)(mean)	LH-BV	Cell count(per ml)(mean)	LH-AV	Cell count(per ml)(mean)	LH-TV
LIVE cell （mean）	6.60666667	LIVE cell （mean）	4.493333333	LIVE cell （mean）	3.8333333	LIVE cell （mean）	7.46
Dead cell （mean）	3.39333333	Dead cell （mean）	5.506666667	Dead cell （mean）	6.1666667	Dead cell （mean）	2.54

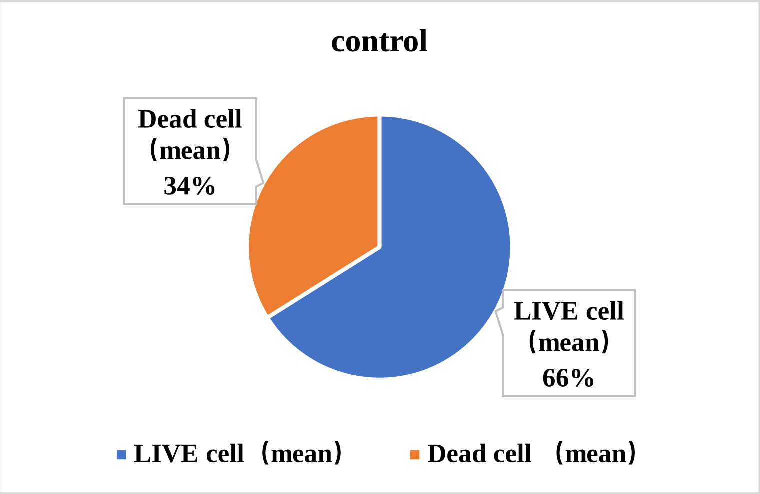

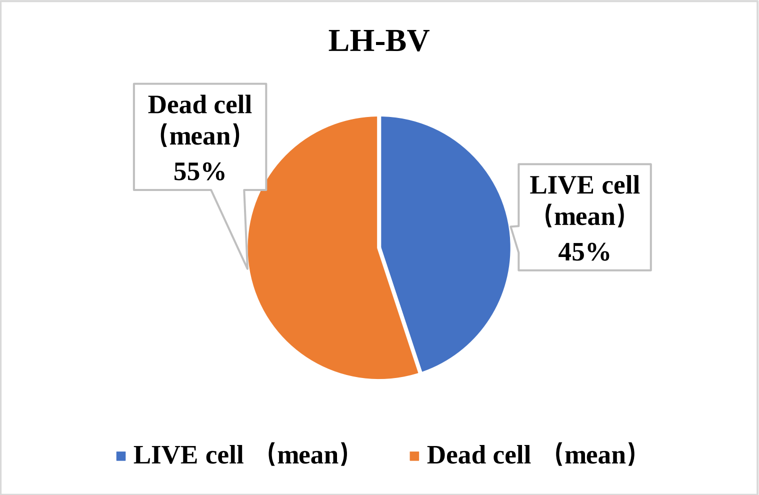

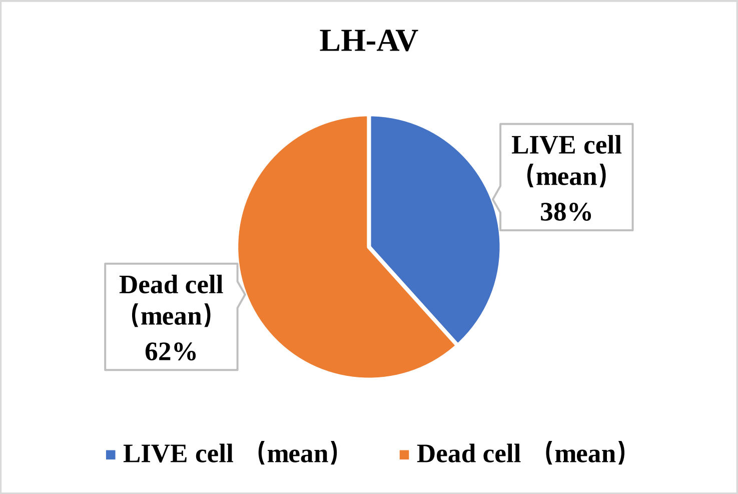

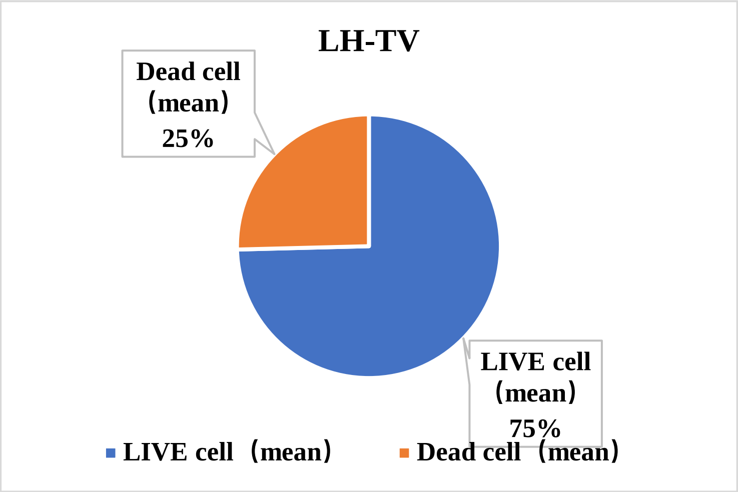

Raw data of Fig 3

Fig3 B				
Number of repetitions	Ki67/Foxl2(mean)	control	Vitrification	LH

1	Fluorescent double label counting	21.722	14.457	30.146
2	Fluorescent double label counting	22.333	14.334	30.567
3	Fluorescent double label counting	20.242	13.232	30.765
Fig3 B (histogram data)				
	Ki67/Foxl2	control	Vitrification	LH
	mean	21.43233	14.007667	30.49267
	SD	1.075175	0.6745564	0.316124

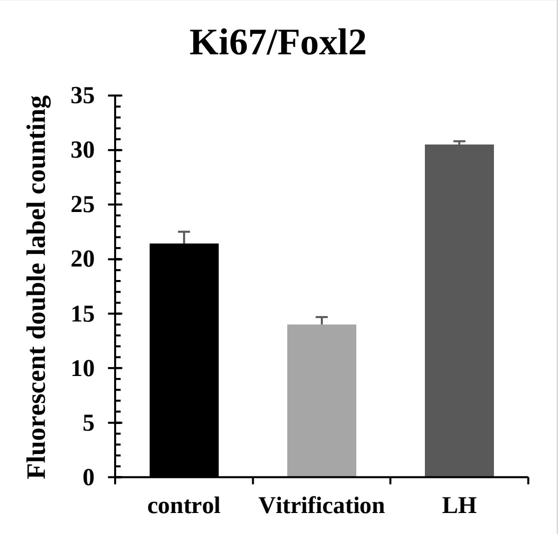

Fig3 D				
Number of repetitions	Ki67/Lgr5(mean)	control	Vitrification	LH
1	Fluorescent double label counting	37.212	23.94	41.377
2	Fluorescent double label counting	38.432	24.111	40.999
3	Fluorescent double label counting	37.456	24.245	41.453
Fig3 D(histogram data)				
	Ki67/Lgr5	control	Vitrification	LH
	mean	37.7	24.098667	41.27633
	SD	0.645563	0.1528736	0.243165

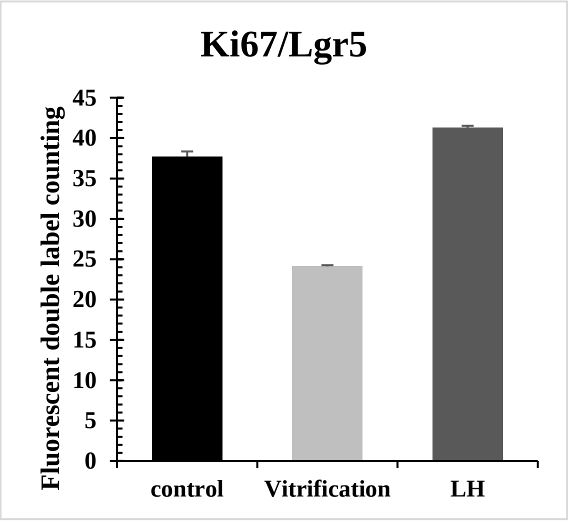

Raw data of Fig 4

Fig4 B				
Number of repetitions	Foxl2(mean)	control	Vitrification	LH
1	Average optical density	1.325	0.702	1.98
2	Average optical density	1.543	0.7344	1.9
3	Average optical density	1.756	0.733	1.899
Fig4 B(histogram data)				
	Foxl2	control	Vitrification	LH
	mean	1.541333	0.723133	1.926333
	SD	0.215505	0.018315	0.046479

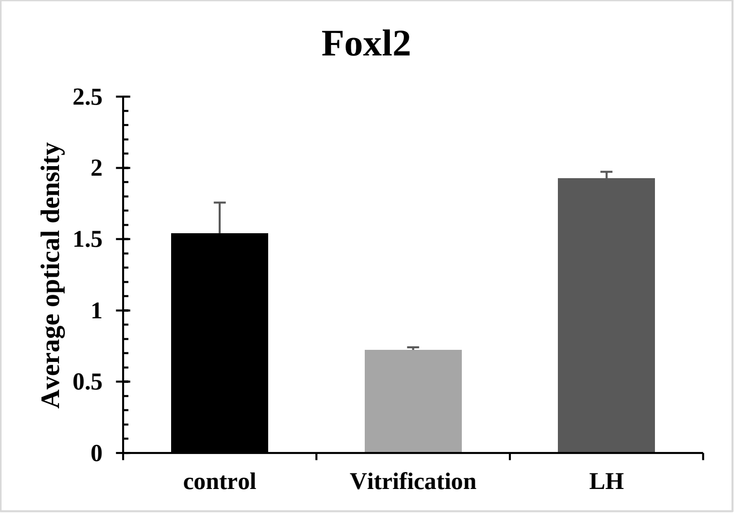

Fig4 D				
Number of repetitions	Foxl2(mean)	control	Vitrification	LH
1	Flourescence intensity of Foxl2	40.155	18.898	57.171
2	Flourescence intensity of Foxl2	39.032	18.154	55.807
3	Flourescence intensity of Foxl2	40.006	17.699	55.028
Fig4 D(histogram data)				
	Foxl2	control	Vitrification	LH
	mean	39.731	18.25033	56.002
	SD	0.609919	0.605277	1.084726

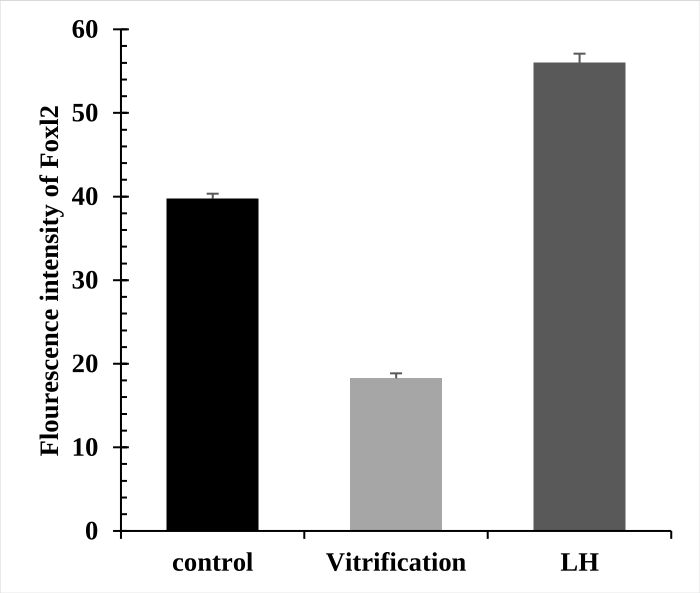

Fig4 F				
Number of repetitions	(mean)	control	Vitrification	LH
1	Foxl2/GAPDH	1.331415	0.636294	2.029759
2	Foxl2/GAPDH	1.605998	0.729908	1.861859
3	Foxl2/GAPDH	1.074492	0.440053	1.30253
Fig4 F(histogram data)				
	Foxl2/GAPDH	control	Vitrification	LH
	mean	1.337302	0.602085	1.731383
	SD	0.265802	0.147925	0.380767

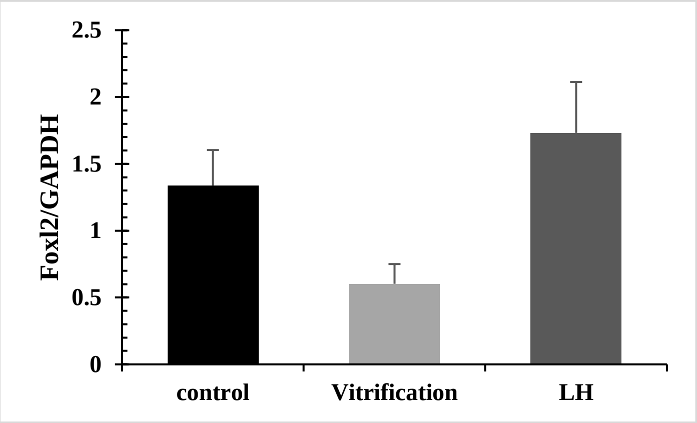

Fig4 G				
Number of repetitions	Foxl2 (mean)	control	Vitrification	LH
1	Relative Foxl2 mRNA expression	0.981686	0.540862	2.075319
2	Relative Foxl2 mRNA expression	1.030492	0.533416	1.023374
3	Relative Foxl2 mRNA expression	0.988514	0.433269	1.709214
Fig4 G(histogram data)				
	Foxl2	control	Vitrification	LH
	mean	1.000231	0.502516	1.602636
	SD	0.026429	0.060085	0.53401

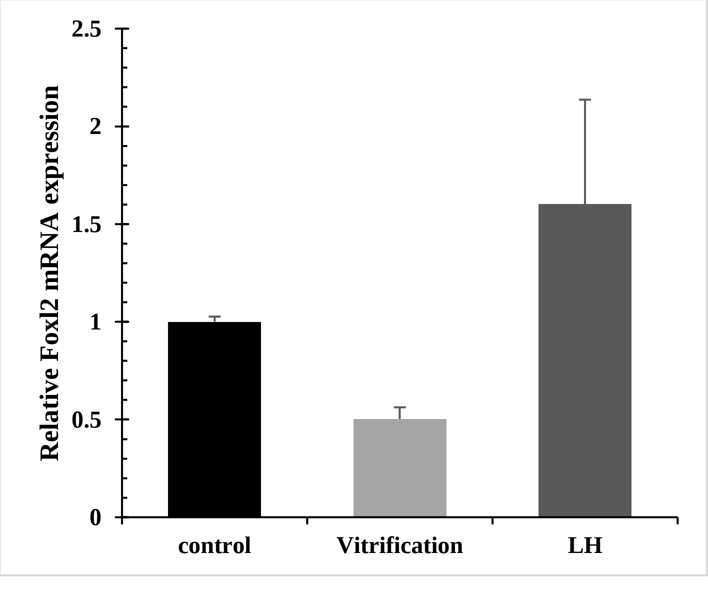

Raw data of Fig 5

Fig5 B				
Number of repetitions	Lgr5(mean)	control	Vitrification	LH
1	Average optical density	1.127751	0.504999	1.406356
2	Average optical density	1.0223	0.50111	1.4789
3	Average optical density	1.034266	0.481376	1.56677
Fig5 B(histogram data)				
	Lgr5	control	Vitrification	LH
	mean	1.061439	0.495828	1.484009
	SD	0.057739	0.012666	0.080329

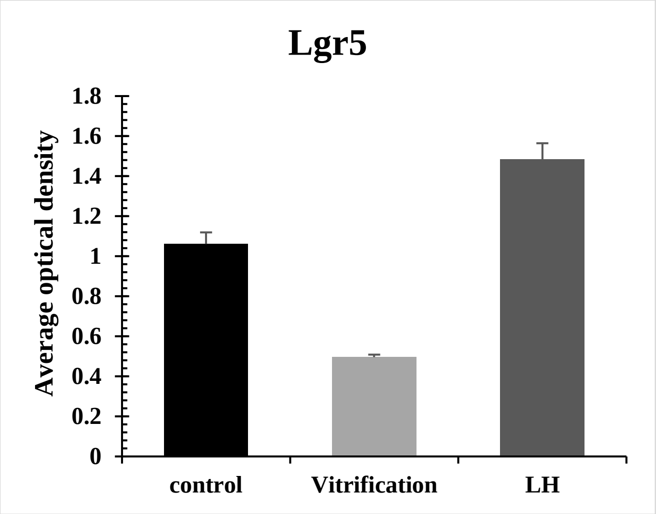

Fig5 D				
Number of repetitions	Lgr5(mean)	control	Vitrification	LH
1	Flourescence intensity of Lgr5	25.801	10.748	35.785
2	Flourescence intensity of Lgr5	25.333	10.567	35.678
3	Flourescence intensity of Lgr5	25.344	10.644	35.765

Fig5 D (histogram data)				
	Lgr5	control	Vitrification	LH
	mean	25.49267	10.653	35.74267
	SD	0.267081	0.090835	0.056889

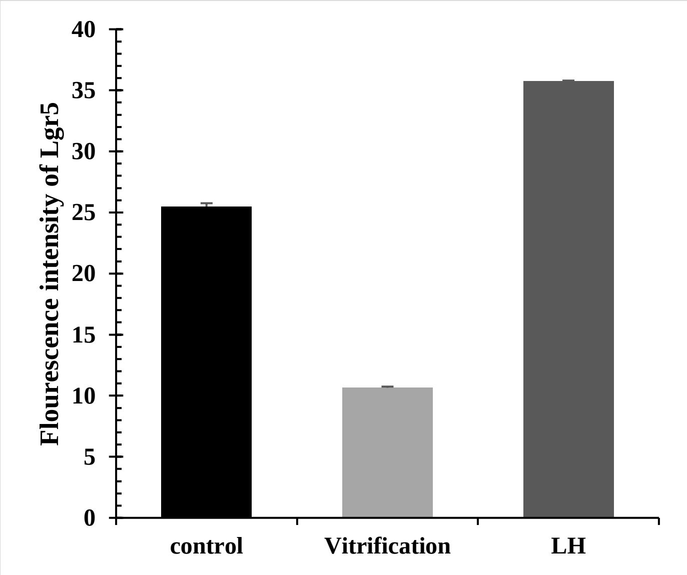

Fig5 F				
Number of repetitions	(mean)	control	Vitrification	LH
1	Lgr5/β−tubulin	0.806114	0.506237	1.197065
2	Lgr5/β-tubulin	1.410711	1.073801	1.609177
3	Lgr5/β-tubulin	1.028681	0.624984	1.230165
Fig5 F(histogram data)				
	Lgr5/β-tubulin	control	Vitrification	LH
	mean	1.081836	0.735007	1.345469
	SD	0.305783	0.299351	0.228977

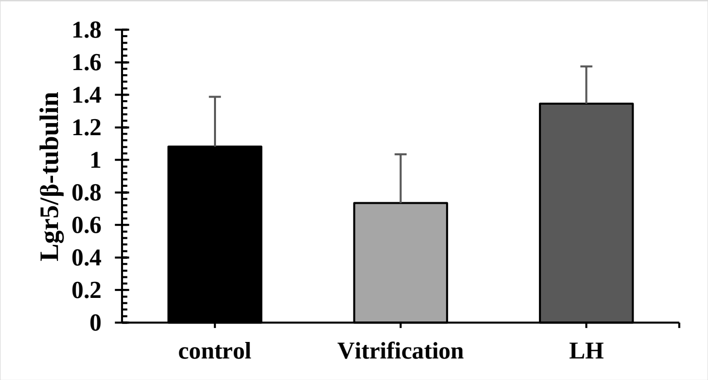

Fig5 G				
Number of repetitions	Lgr5 (mean)	control	Vitrification	LH

1	Relative Lgr5 mRNA expression	0.856584	0.20829	0.918064
2	Relative Lgr5 mRNA expression	1.076738	0.234339	0.905425
3	Relative Lgr5 mRNA expression	1.084227	0.152477	0.756109
Fig5 G(histogram data)				
	Lgr5	control	Vitrification	LH
	mean	1.005849	0.198369	0.859866
	SD	0.129322	0.041823	0.090078

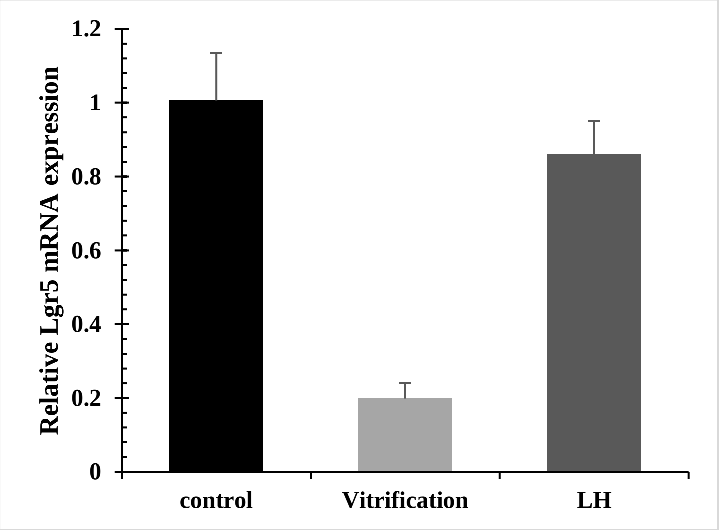

Raw data of Fig 6

Fig6 B				
Number of repetitions	LHR(mean)	control	Vitrification	LH
1	Flourescence intensity of LHR	35.247	31.45	48.123
2	Flourescence intensity of LHR	35.126	31.46687	48.533
3	Flourescence intensity of LHR	34.342	31.2456	48.754
Fig6 B (histogram data)				

	LHR	control	Vitrification	LH
	mean	34.905	31.38749	48.47
	SD	0.491312	0.123169	0.320183

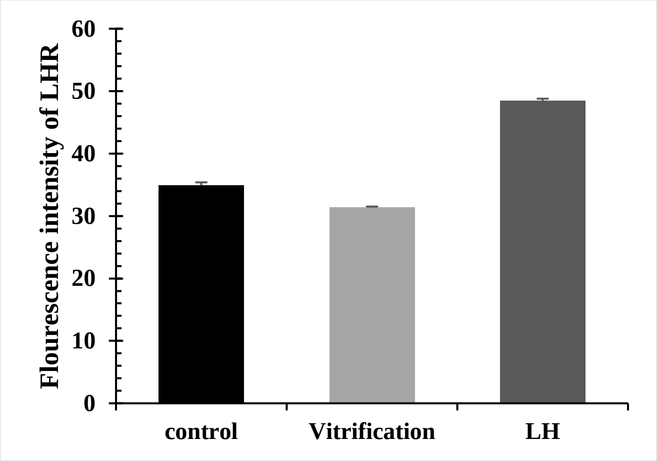

Fig6 C				
Number of repetitions	LHR (mean)	control	Vitrification	LH
1	Relative LHR mRNA expression	0.885031	1.055993	1.593943
2	Relative LHR mRNA expression	0.943177	0.738976	1.509008
3	Relative LHR mRNA expression	1.197977	0.726281	1.690676
Fig6 C(histogram data)				
	LHR	control	Vitrification	LH
	mean	1.008728	0.840417	1.597875
	SD	0.166453	0.186802	0.090898

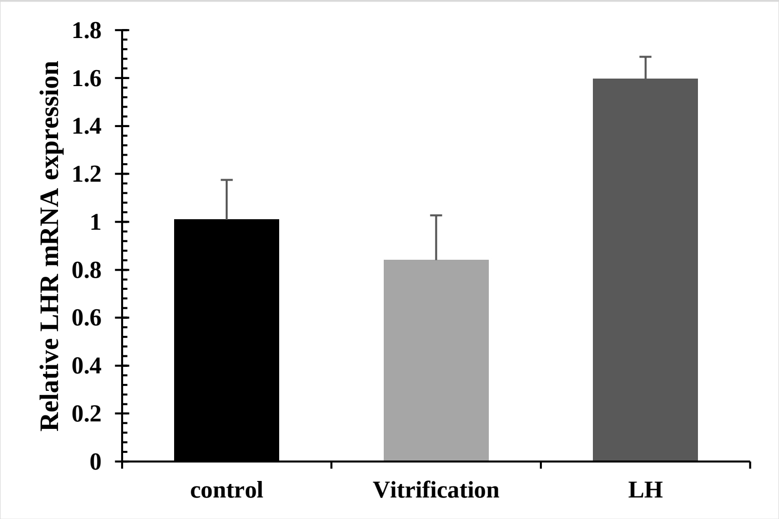

Fig6 E				
Number of repetitions	(mean)	control	Vitrification	LH
1	LHR/GAPDH	0.830473	0.722659	2.235727
2	LHR/GAPDH	1.551425	1.205125	1.675369
3	LHR/GAPDH	0.767374	0.709111	0.893348
Fig6 E(histogram data)				
	LHR/GAPDH	control	Vitrification	LH
	mean	1.049757	0.878965	1.601481
	SD	0.435601	0.282544	0.674233

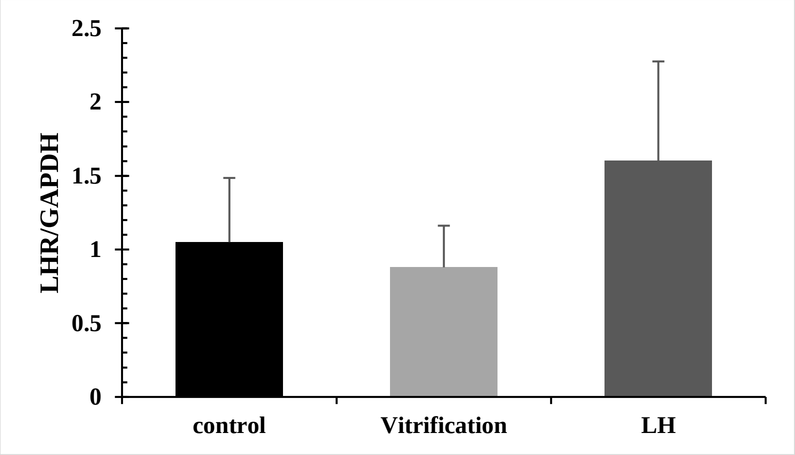

Raw data of Fig 7

Fig7 C				
Number of repetitions	active caspase-3(mean)	control	Vitrification	LH
1	Average optical density	0.59894	0.904974	0.29213
2	Average optical density	0.589388	0.96567	0.30222
3	Average optical density	0.59257	0.936788	0.29999
Fig7 C(histogram data)				
	active caspase-3	control	Vitrification	LH
	mean	0.593633	0.935811	0.298113
	SD	0.004864	0.03036	0.0053

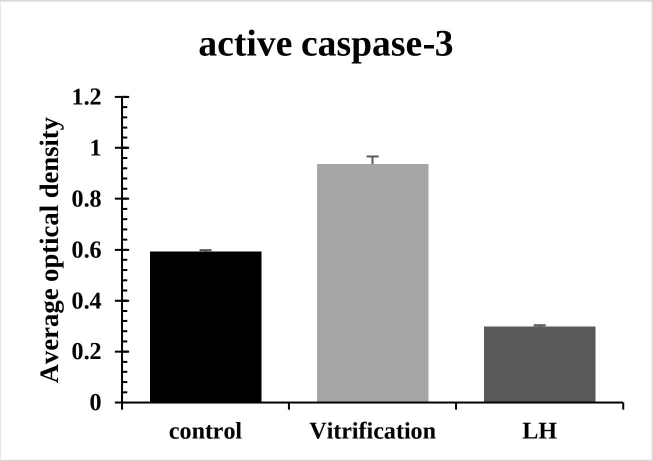

Fig7 E				
Number of repetitions	(mean)	control	Vitrification	LH
1	active caspase-3/β-tubulin	1.052731	1.417763	0.426131
2	active caspase-3/β-tubulin	0.64468	1.290868	0.451748
3	active caspase-3/β-tubulin	1.100343	1.224178	0.716031
Fig7 E(histogram data)				
	active caspase-3/β-tubulin	control	Vitrification	LH
	mean	0.932585	1.310936	0.531303
	SD	0.250467	0.09834	0.160491

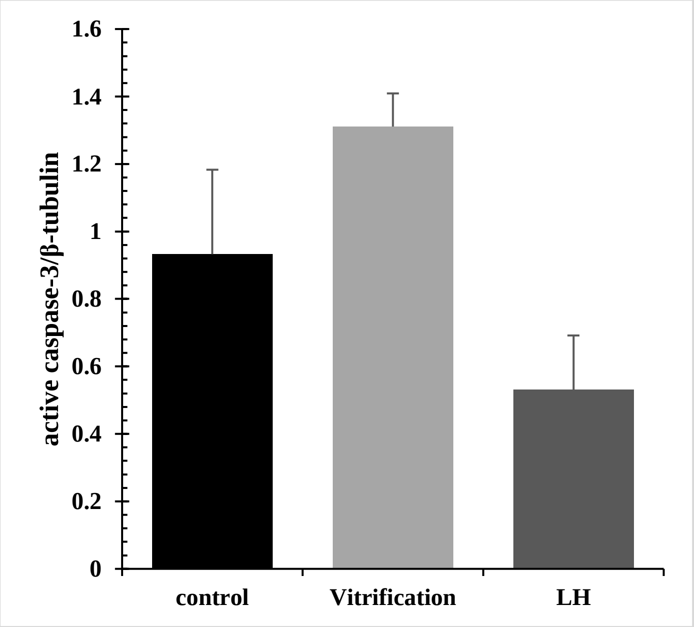

Fig7 F				
Number of repetitions	active caspase-3 (mean)	control	Vitrification	LH
1	Relative active caspase-3 mRNA expression	1.526259	3.482202	1.274561
2	Relative active caspase-3 mRNA expression	0.817902	3.434262	1.028114
3	Relative active caspase-3 mRNA expression	0.80107	3.07375	1.109569
Fig7 F(histogram data)				
	active caspase-3	control	Vitrification	LH
	mean	1.04841	3.330071	1.137415
	SD	0.413915	0.223271	0.125561

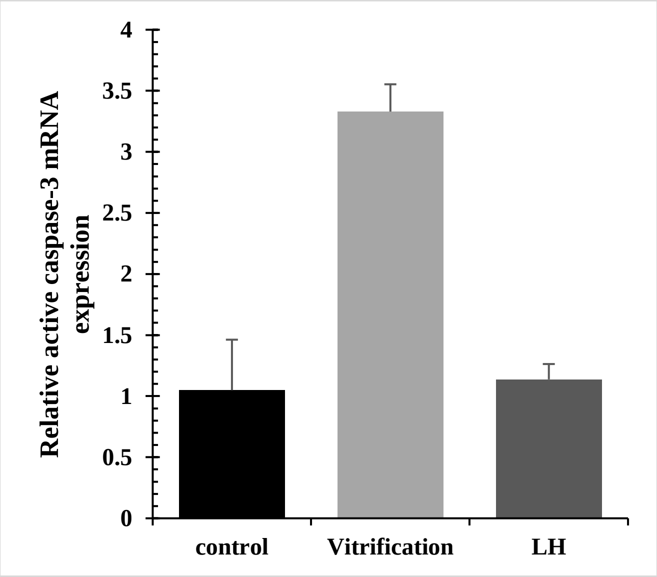

Raw data of Fig 8

Fig8 B TGF-β				
Number of repetitions	TGF-β(mean)	control	Vitrification	LH
1	Average optical density	1.732495	1.402398	2.893395

2	Average optical density	1.67888	1.4988	2.899
3	Average optical density	1.699	1.4899	2.7788
Fig8 B(histogram data)				
	TGF-β	control	Vitrification	LH
	mean	1.703458	1.463699	2.857065
	SD	0.027084	0.053275	0.067837

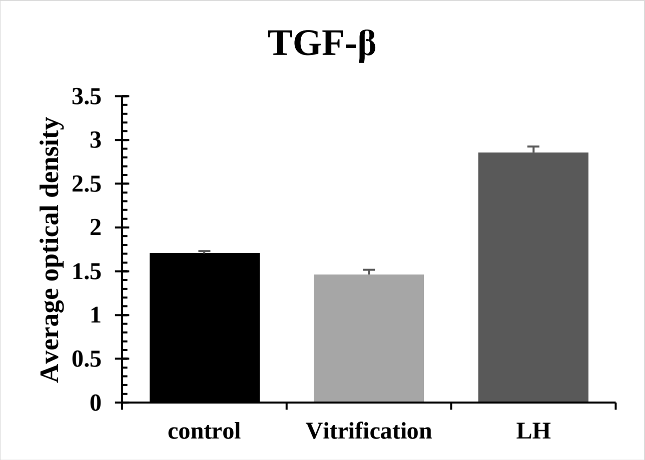

Fig8 C GDF-9				
Number of repetitions	GDF-9(mean)	control	Vitrification	LH
1	Average optical density	1.877512	0.594999	2.406356
2	Average optical density	1.7876	0.5446	2.43436
3	Average optical density	1.7554	0.7688	2.3563
Fig8 C(histogram data)				
	GDF-9	control	Vitrification	LH
	mean	1.806837	0.636133	2.399005
	SD	0.063288	0.117624	0.039546

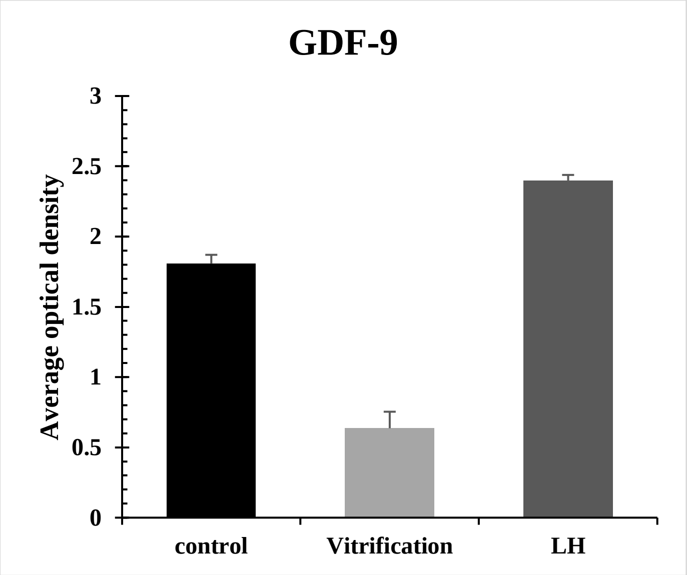

Fig8 D BMP-15				
Number of repetitions	BMP-15(mean)	control	Vitrification	LH
1	Average optical density	2.09894	0.604974	3.19213
2	Average optical density	2.05848	0.6146	3.156989
3	Average optical density	2.05456	0.6755	3.18097
Fig8 D(histogram data)				
	BMP-15	control	Vitrification	LH
	mean	2.07066	0.631691	3.176696
	SD	0.024569	0.038243	0.017956

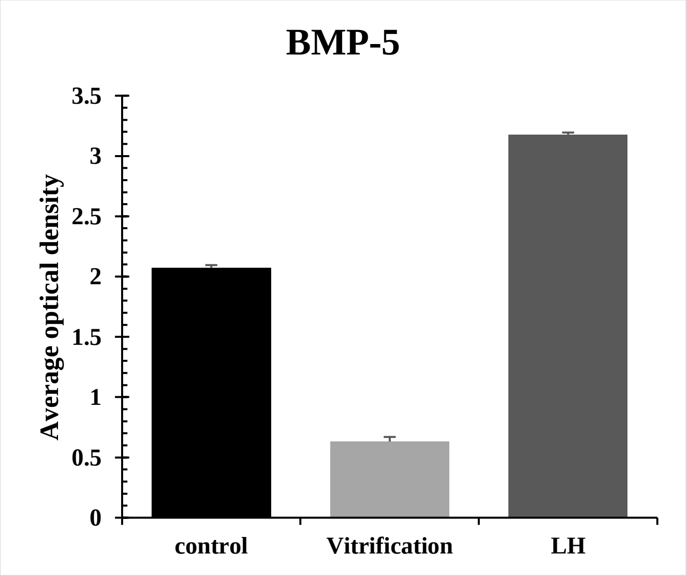

Fig8 H				
Number of repetitions	(mean)	control	Vitrification	LH
1	TGF-β/GAPDH	1.141904	0.787985	1.45871
2	TGF-β/GAPDH	0.951538	0.706035	1.149047
3	TGF-β/GAPDH	1.008549	0.887297	0.73416
Fig8 H(histogram data)				
	TGF-β/GAPDH	control	Vitrification	LH
	mean	1.033997	0.793772	1.113973
	SD	0.097701	0.09077	0.363547

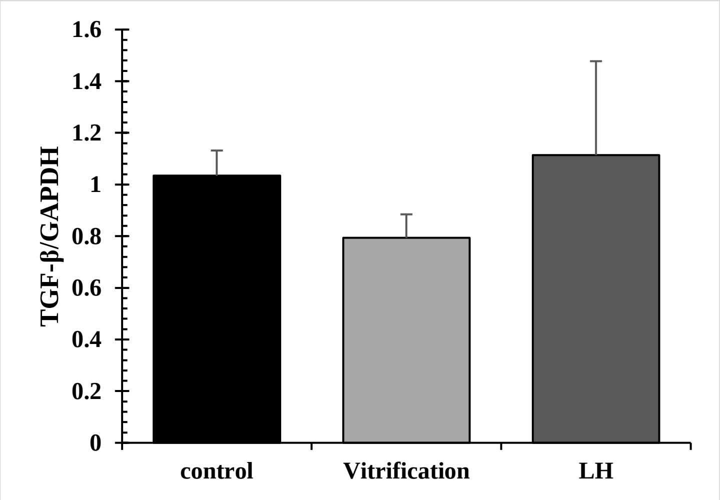

Fig8 I				
Number of repetitions	(mean)	control	Vitrification	LH
1	GDF-9/GAPDH	2.139305	1.84549	2.692529
2	GDF-9/GAPDH	2.42379	1.779011	3.438699
3	GDF-9/GAPDH	2.491792	2.004662	2.368425
Fig8 I(histogram data)				
	GDF-9/GAPDH	control	Vitrification	LH
	mean	2.351629	1.876388	2.833218
	SD	0.186995	0.115955	0.548832

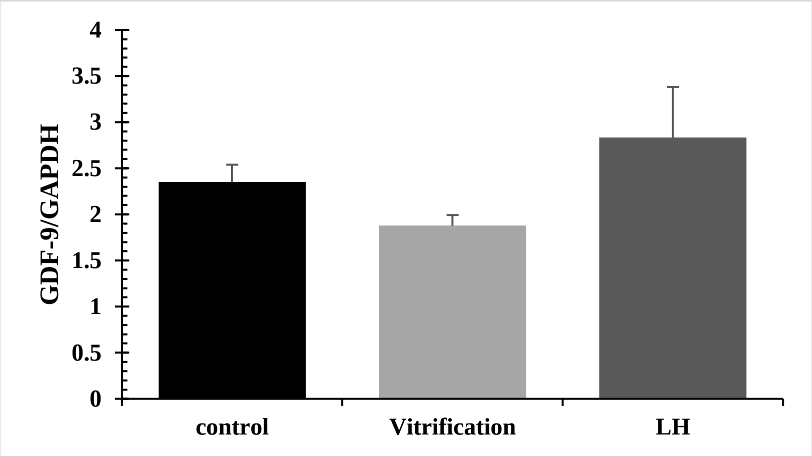

Fig8 J				
Number of repetitions	(mean)	control	Vitrification	LH
1	BMP-15/GAPDH	2.315921	1.309585	2.418856
2	BMP-15/GAPDH	1.631411	0.998599	1.897403
3	BMP-15/GAPDH	1.511717	1.437835	1.943528
Fig8 J(histogram data)				
	BMP-15/GAPDH	control	Vitrification	LH
	mean	1.819683	1.248673	2.086596
	SD	0.433902	0.225865	0.288669

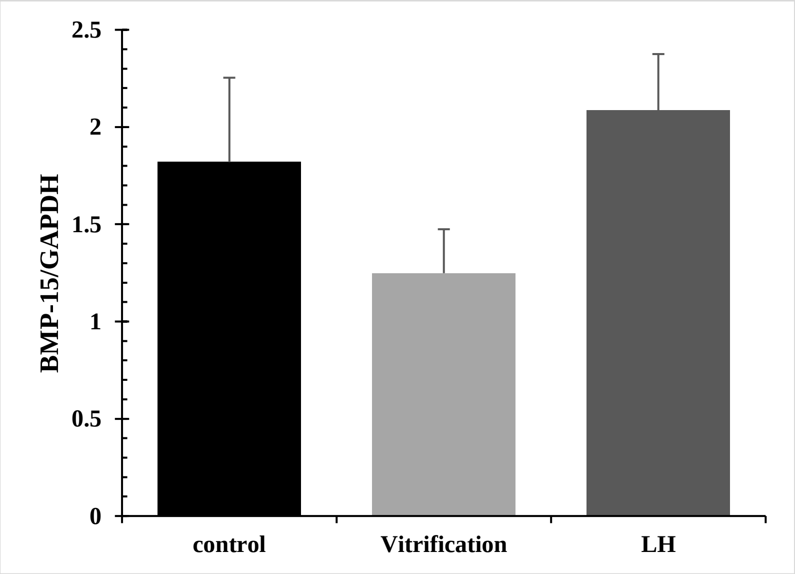

Fig8 K				
Number of repetitions	TGF-β (mean)	control	Vitrification	LH

1	Relative TGF-β mRNA expression	0.97942	0.570382	1.328686
2	Relative TGF-β mRNA expression	1.049717	0.479632	1.239708
3	Relative TGF-β mRNA expression	0.972655	0.566442	2
Fig8 K(histogram data)				
	TGF-β	control	Vitrification	LH
	mean	1.000597	0.538819	1.522798
	SD	0.042673	0.051295	0.415657

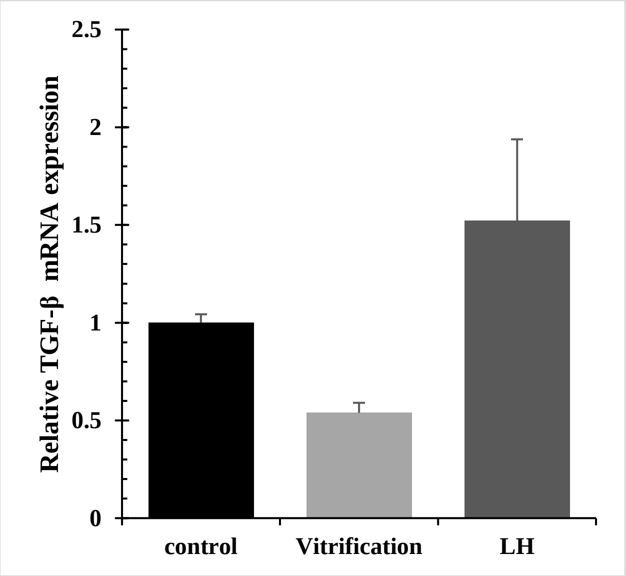

Fig8 L				
Number of repetitions	GDF-9 (mean)	control	Vitrification	LH
1	Relative GDF-9 mRNA expression	0.935191	0.279968	1.76949
2	Relative GDF-9 mRNA expression	1.135504	0.595979	1.977028
3	Relative GDF-9 mRNA expression	0.941696	0.457973	1.697408
Fig8 L(histogram data)				
	GDF-9	control	Vitrification	LH

	mean	1.004131	0.44464	1.814642
	SD	0.11382	0.158427	0.145175

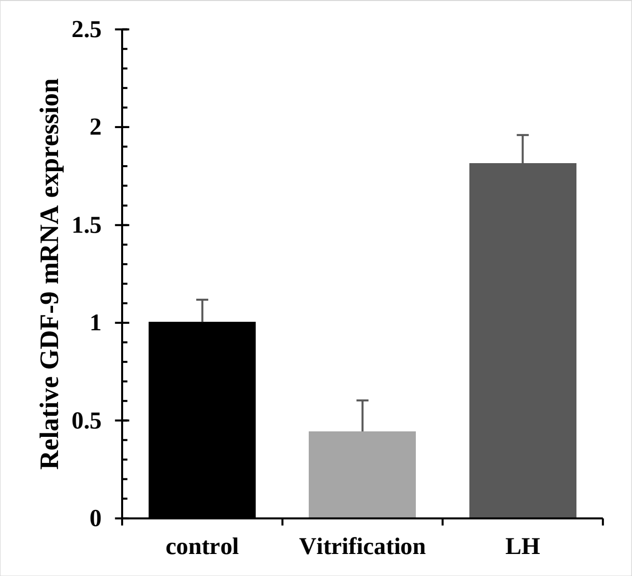

Fig8 M				
Number of repetitions	BMP-15(mean)	control	Vitrification	LH
1	Relative BMP-15 mRNA expression	0.988514	0.608502	1.304352
2	Relative BMP-15 mRNA expression	1.052145	0.529732	0.814131
3	Relative BMP-15 mRNA expression	0.961483	0.51883	0.718636
Fig8 M(histogram data)				
	BMP-15	control	Vitrification	LH
	mean	1.000714	0.552354	0.945706
	SD	0.046546	0.04893	0.314245

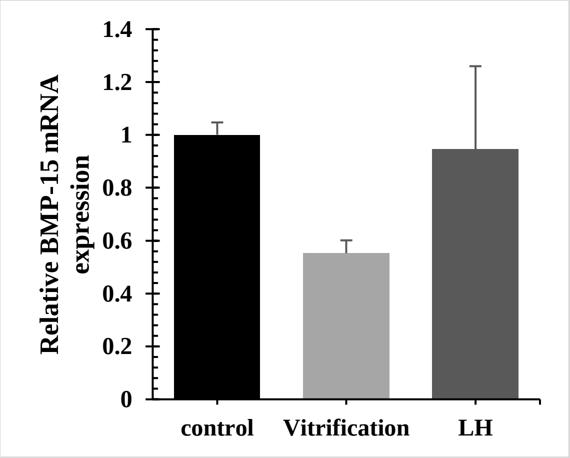

Supplement: S2 File — (PDF) [file pone.0317416.s002.pdf]
