# Supplementary material for: Protective effect of luteinizing hormone on frozen-thawed ovarian follicles and granulosa cells
Source: PLoS One. 2025 Jan 14;20(1):e0317416. doi: 10.1371/journal.pone.0317416 (PMC11731763; doi:10.1371/journal.pone.0317416)
Supplement: Attachment — Submitted filename: Response to Reviewers.docx [file pone.0317416.s003.docx]

**Responses to the** **Journal’ Comments**

***1.*** ***When submitting your revision, we need you to address these additional requirements.***

***Please ensure that your manuscript meets PLOS ONE's style requirements, including those for file naming. The PLOS ONE style templates can be found at***

***https://journals.plos.org/plosone/s/file?id=wjVg/PLOSOne_formatting_sample_main_body.pdf and***

***https://journals.plos.org/plosone/s/file?id=ba62/PLOSOne_formatting_sample_title_authors_affiliations.pdf.***

**Response:**

We sincerely appreciate the time you have dedicated to reviewing our manuscript. We are well aware of the importance of the style requirements of POLS ONE, and after carefully reading “PLOS Affiliations Formatting Guidelines” and “PLOS Manuscript Body Formatting Guidelines”, We edited the manuscript in strict accordance with the requirements. Expect to be as style requirements perfect as possible.

***2. PLOS ONE now requires that authors provide the original uncropped and unadjusted images underlying all blot or gel results reported in a submission’s figures or Supporting Information files. This policy and the journal’s other requirements for blot/gel reporting and figure preparation are described in detail at https://journals.plos.org/plosone/s/figures#loc-blot-and-gel-reporting-requirements and https://journals.plos.org/plosone/s/figures#loc-preparing-figures-from-image-files. When you submit your revised manuscript, please ensure that your figures adhere fully to these guidelines and provide the original underlying images for all blot or gel data reported in your submission. See the following link for instructions on providing the original image data: https://journals.plos.org/plosone/s/figures#loc-original-images-for-blots-and-gels.***

***In your cover letter, please note whether your blot/gel image data are in Supporting Information or posted at a public data repository, provide the repository URL if relevant, and provide specific details as to which raw blot/gel images, if any, are not available. Email us at plosone@plos.org if you have any questions.***

**Response:**

Thank you for your patient guidance on how to submit the original blot images and the specific details. We have carefully marked as required, and uploaded all the original image data related to the gel images in the manuscript in the form of Supporting information to ensure compliance with the requirements of the journal.

***3.*** ***To comply with PLOS ONE submissions requirements, in your Methods section, please provide additional information regarding the experiments involving animals and ensure you have included details on (1) methods of sacrifice, (2) methods of anesthesia and/or analgesia, and (3) efforts to alleviate suffering.***

**Response:**

Thank you for your efforts to make our manuscript more compliant with journal requirements. As requested, we have added methods of sacrifice and efforts to alleviate the suffering of mice in Ethics statement of manuscript, and that methods of anesthesia and/or analgesia steps were avoided because there was no surgery or modeling in this study. The details are on page 6, lines 111-113, and marked them in red.

***4.*** ***Thank you for stating the following financial disclosure:***

***[This study was funded by the Natural and Science Foundation of Inner Mongolia Autonomous Region (No. 2019MS08139, 2022QN03003).].***

***Please state what role the funders took in the study. If the funders had no role, please state: ""The funders had no role in study design, data collection and analysis, decision to publish, or preparation of the manuscript.""***

***If this statement is not correct you must amend it as needed.***

***Please include this amended Role of Funder statement in your cover letter; we will change the online submission form on your behalf.***

**Response:**

Thank you for your careful consideration of the efforts that funders put into the research. The role of the funder in this study is consistent with what the author filled out in the submission system at the time of the initial submission, as follows:

Role of Funder statement: Jie Chen: Conceptualization, Formal Analysis, Funding acquisition, Visualization, Writing - Original Draft, Writing - Review & Editing. Boyang Yu: Conceptualization, Funding acquisition, Investigation, Validation, Writing - Review & Editing.

***5. We note that your Data Availability Statement is currently as follows: [All relevant data are within the manuscript and its Supporting Information files.]***

***Please confirm at this time whether or not your submission contains all raw data required to replicate the results of your study. Authors must share the “minimal data set” for their submission. PLOS defines the minimal data set to consist of the data required to replicate all study findings reported in the article, as well as related metadata and methods (https://journals.plos.org/plosone/s/data-availability#loc-minimal-data-set-definition).***

***For example, authors should submit the following data:***

***- The values behind the means, standard deviations and other measures reported;***

***- The values used to build graphs;***

***- The points extracted from images for analysis.***

***Authors do not need to submit their entire data set if only a portion of the data was used in the reported study.***

***If your submission does not contain these data, please either upload them as Supporting Information files or deposit them to a stable, public repository and provide us with the relevant URLs, DOIs, or accession numbers. For a list of recommended repositories, please see https://journals.plos.org/plosone/s/recommended-repositories.***

***If there are ethical or legal restrictions on sharing a de-identified data set, please explain them in detail (e.g., data contain potentially sensitive information, data are owned by a third-party organization, etc.) and who has imposed them (e.g., an ethics committee). Please also provide contact information for a data access committee, ethics committee, or other institutional body to which data requests may be sent. If data are owned by a third party, please indicate how others may request data access.***

**Response:**

Thank you for your patient guidance on the scope, content, method, etc. of submitting raw data. The original data of all experimental results involved in the manuscript have been uploaded as supporting information in a "minimum data set" PDF file, and there are no ethical or legal restrictions on all data in this paper.

***6. We note that*** ***Figure(s) 2, 3, 4, 5, 6, 7, and 8 in your submission contain copyrighted images. All PLOS content is published under the Creative Commons Attribution License (CC BY 4.0), which means that the manuscript, images, and Supporting Information files will be freely available online, and any third party is permitted to access, download, copy, distribute, and use these materials in any way, even commercially, with proper attribution. For more information, see our copyright guidelines: http://journals.plos.org/plosone/s/licenses-and-copyright.***

***We require you to either (1) present written permission from the copyright holder to publish these figures specifically under the CC BY 4.0 license, or (2) remove the figures from your submission: a. You may seek permission from the original copyright holder of Figure(s) 2, 3, 4, 5, 6, 7, and 8 to publish the content specifically under the CC BY 4.0 license.***

***We recommend that you contact the original copyright holder with the Content Permission Form (http://journals.plos.org/plosone/s/file?id=7c09/content-permission-form.pdf) and the following text:“I request permission for the open-access journal PLOS ONE to publish XXX under the Creative Commons Attribution License (CCAL) CC BY 4.0 (http://creativecommons.org/licenses/by/4.0/). Please be aware that this license allows unrestricted use and distribution, even commercially, by third parties. Please reply and provide explicit written permission to publish XXX under a CC BY license and complete the attached form.”***

***Please upload the completed Content Permission Form or other proof of granted permissions as an ""Other"" file with your submission.***

***In the figure caption of the copyrighted figure, please include the following text: “Reprinted from [ref] under a CC BY license, with permission from [name of publisher], original copyright [original copyright year].”***

***b. If you are unable to obtain permission from the original copyright holder to publish these figures under the CC BY 4.0 license or if the copyright holder’s requirements are incompatible with the CC BY 4.0 license, please either i) remove the figure or ii) supply a replacement figure that complies with the CC BY 4.0 license. Please check copyright information on all replacement figures and update the figure caption with source information. If applicable, please specify in the figure caption text when a figure is similar but not identical to the original image and is therefore for illustrative purposes only.***

**Response:**

The strict copyright requirements of journals are the precursor guarantee for the publication of the manuscript. Thank you for your guidance in this regard. According to the requirements of the journal, we decided to delete the copyright images in the figure(s) 2, 3, 4, 5, 6, 7, and 8. At the same time, we used the original data to re-create the corresponding histogram of each figure in the Excel. We believe that the above methods can avoid copyright problems, but if our modifications are still not perfect, please indicate that we will further correct.

***7.Please include your tables as part of your main manuscript and remove the individual files. Please note that supplementary tables (should remain/ be uploaded) as separate "supporting information" files.***

**Response:**

Thank you for your precise guidance on how to insert the table as the requirments of PLOS ONE. We have placed the tables included directly after the paragraph in which they are first cited, while deleting the individual file where the tables were written. It is believed that inserting the form in this way meets the requirements of the journal.

***8. Please upload a copy of*** ***Supplementary*** ***Figure 1, Supplementary Figure 2, Supplementary Figure 3 and Supplementary Figure 4 to which you refer in your text on page 37 and 38. Please amend the file type to 'Supporting Information'. If the Supplementary file is no longer to be included as part of the submission please remove all reference to it within the text.***

**Response:**

With your patient guidance, We have removed the text on pages 37 and 38 of the original manuscript "Supplementary Figure 1”, “Supplementary Figure 2”, “Supplementary Figure 3” and “Supplementary Figure 4 ". At the same time, according to the requirements of the journal, all the original data of the blot pictures have been uploaded in the form of supporting information.

**Responses to the Reviewers’ Comments**

**Responses to the Comments of Reviewer 1:**

***1. In Figure 2, the authors have shown results of Live/ Dead cells only for the LH TV category. Although the morphology for LH-BV and LH-AV were reported to be different from control and LH-TV cells, the Live/ Dead cells ratio for all the categories tested will help in understanding the scenario better.***

**Response:**

We sincerely thank you for taking the time to review the manuscript and for giving us the greatest encouragement and very important suggestions. Obviously, the Live/ Dead cells ratio for all the categories tested will help in understanding the scenario better. We took your advice and added Live/Dead detection experiments of all groups and the results have been supplemented in the Fig 2 B-F of munuscript. At the same time, we also have stronger evidence for the experimental results.

***2.Upregulation/ downregulation of Foxl2, LGR5, LHR, Caspase-3, were reported at protein level by Western Blot. Is there any specific reason why Western Blot not done for TGF -β and GDF-9 and BMP-15. (Figure 8)***

**Response:**

Thank you very much for your careful review of this part of the experiment. Although we have completed the protein imprinting detection of granular cell specific expression factors, we still ignore the importance of protein detection in the aspect of signaling pathway. Therefore, according to your comments, we carefully supplemented the western blot detection of TGF-β, GDF-9 and BMP-15 in Fig 8 E-J of the manuscript. We ensured that the experimental methods and steps were consistent with the previous experiments, at the same time, the expression of target gene was further verified.

**Minor Comment:**

***The axis labels are not visible clearly for figure 4B.***

**Response:**

Thank you for your accurate feedback on Figure 4B, and we apologize for the serious problems caused by the typographical error. At your suggestion, we have edited Fig 4 B to make sure everything is clearly visible.

**Responses to the Comments of Reviewer 2:**

Thank you very much for your careful review and kind comments on our manuscript. Although we wrote the paper very carefully and repeatedly adjusted the language, there are still many mistakes in our expression and language structure that need to be corrected. Your comments definitely help us improve the quality of this manuscript. We have revised it point-by-point according to your comments as follows.

***1.*** ***I understand the author’s effort to make the methods easy. However, the structure and language need significant improvement; for example, the abstract could be more precise and concise. The materials and methods need proper editing to be meaningful, e.g., “Reagent preparation: thawing reagent, restore to room temperature, equipped with 1x working solution, now in use.” What do all these mean? What do LA and LB mean? “An is 2 μM”?***

**Response:**

We appreciate your valuable reminder to comments on the structure of the language, and we apologize for not precise and concise enough of the abstract and for the inaccurate language in the material methodology.

In our revised manuscript, we have made specific modifications to the above problems and marked them in red, The details are as follows:

①We rewrote the abstract to make the description as precise and concise as possible.

②The materials and methods section has been re-edited, we have replaced the “Reagent preparation: thawing reagent, restore to room temperature, equipped with 1x working solution, now in use.” with “The process involved thawing the necessary reagents, followed by vigorous vortexing to mix 30 µL of 1.5 mM PI with 5 µL of 4mM Calcein AM with in a 10 mL solution of PBS, obtained from Seven, Beijing, China.” in the line of 169-171 of the revised munuscript.

③“LA, LB and 2 μ M ” in the original manuscript has been deleted in the revised munuscript.

***Materials and Methods:***

***2.7 does not make reading any easier. For example,*** ***“the second first antibody was added.”***

**Response:**

In 2.7 of the the revised manuscript, we have replaced the“the second first antibody was added.” with “A second round of antigen retrieval preceded the addition of either rabbit-derived monoclonal antibodies against Foxl2 (1:200, abcam, UK) or Lgr5 (1:1000, abcam, UK), which were also incubated overnight at 4 °C.” in the line of 187-189 on page 9 of the revise manuscript.

***2.8 the same group was mixed and randomly divided into holes.***

**Response:**

“the same group was mixed and randomly divided into holes.” in 2.8 in the original manuscript has been deleted in the revised manuscript.

***2.9, “After allowing the cells to settle in the counting pool and fill the entire grid, counting commenced.”***

***These are a few examples, but they are to imply the seriousness of thorough editing.***

**Response:**

“After allowing the cells to settle in the counting pool and fill the entire grid, counting commenced.” in 2.9 has been deleted in revised manuscript.

In addition to the above specific errors you raised, we have also tried to correct other misrepresentations in the entire article, such as: we have replaced the “In the first experiment” in line 140 on page 7 of the original manuscript with “In the study examining optimal LH intervention protocols” in line 126 on page 6 of the revised manuscript. In addition, we have replaced the "1:0" in line 259 on page 12 of the original manuscript with "1:200" in line 233 on page 11 of the revised manuscript. And the above changes are highlighted in red. We have tried our best to correct such errors in the revised manuscript and hope to meet your requirements. If there are still any shortcomings after revision, please raise them and we will correct them. Your rigorous scientific attitude has set an example for us.

***2.14*** ***“1ul of cDNA”: Please avoid using this kind of terminology in any scientific manuscript; instead, please add the amount of RNA*** ***used for reverse transcription.***

**Response:**

“1ul of cDNA” in 2.14 in the original manuscript has been deleted. And“3 μL RNA used for reverse transcription” has been added in 2.13 in the revised manuscript line 261.

***2. The Tiff image for Fig 2 is unavailable, and it’s too challenging to read Fig 2 B. Many of the figure labels and Tiff files are not labeled, e.g., F. I don’t know whether I'm missing something or if the journal or the authors missed it. Where are the tables in the document?***

**Response:**

We appreciate your feedback regarding on the formatting and labeling issues of Fig 2, in particular Fig 2B and the missing table. We apologize for any confusion caused by the lack of annotations and any unclear instructions in the images. We value your advice and have taken these issues into account in the revised manuscript.

To make sure that the updated figures are easier to detect and read, we performed the experiments and replaced Fig 2 B. This will make it possible for readers to judge the size and proportions of the structures clearly. And the Fig 2 was re-stored in TIFF format in Photoshop as required.

The table 2 was inserted in 3.1, the line 307 in the revised manuscript.

***3.*** ***Figure 3 Authors implied that proliferative markers Ki67*** ***co-localize with Fox12 and Lgr5. I agree with the observation, but why is the proliferation marker granular compared with the more*** ***distributed Fox12 and Lgr5? Table 3 is mentioned as missing.***

**Response:**

Thank you for your approval of the co-localize results in Figure 3, and thank you very much for your very professional questions about Figure 3. We are very sorry for the confusion caused by not inserting table3 into the manuscript when submitting the paper for the first time.

We have carefully considered the questions you raised that are of great value to the results of this study. According to literature reports, Foxl2 and Lgr5 are widely expressed in different types of granular cells, while Ki67 is mainly expressed in proliferating cells, so the positive expression of Ki67 is more dispersed.

At the same time, we have added discussion result in the discussion of the revised manuscript lines 454-457. The reference 32 for the above discussion is newly cited.

The table 3 has inserted in 3.2, the line 335 in the revised manuscript.

***4. The figures’ representation and clarity are commendable; however, notable errors are present, such as the overlap between Fig 4 D and B.Figure “Real-time PCR detection of Foxl2 mRNA revealed pronouncedly higher expression in the LH group compared with in the control and vitrification groups (P<0.05) (Fig.4F). That is not true from the graph. It is almost twofold less than control. This raises the question of how protein levels are higher for Foxl2 in LH. Is there any translational regulation, protein stability, or mRNA stability? It needs an explanation from the authors. While controlled overexpression might be helpful for FoxL2 function, unregulated or excessive overexpression could harm granulosa cells and ovarian function. Please have a counterargument.***

**Response:**

Thank you for your comments on Fig 4. We attach great importance to your comments and try our best to make improvements according to your guidance. If the revised result is still insufficient due to our misunderstanding, please raise it again, and we guarantee to correct it according to your comments.

In order to correct the overlap between Fig 4 D and B, we reformatted all the resulting images in Fig 4 to make the layout as neat and scientific as possible while ensuring the requirements of the journal submission. In order to verify the expression of Foxl2 mRNA, we repeated the experiment three times, and the results were consistent with the expression results of Foxl2 protein. There may have the probloms of mRNA instability in the past experiments in Fig 4 F. In order to avoid the damage of Foxl2 overexpression on granulosa cells and ovarian function, we selected the optimal intervention concentration of LH, namely 0.3IU/mL, according to the results of the previous experiment to complete the experiment. According to our previous experimental studies, the concentration of 0.3 IU/mL is the best dose of LH intervention, and the test results are the most favorable for all aspects of the ovary.

***5. Figure 6D. The authors concluded the results: “The results of Real-time PCR detection confirmed the localization and quantitative expression of LHR, which showed that from high to low of the expression of LHR mRNA was LH group, control group, and vitrification group (P<0.05) (Fig.6D). Can a “Real-time PCR” confirm mRNA localization? The latter part is just words. Randomly put it as a sentence!***

**Response:**

Thank you for correcting the description of Figure 6D. Your accuracy in describing scientific problems and rigorous scientific attitude set an example for us. We carefully corrected our mistakes and understood the importance of accurately describing scientific problems.

In 3.4 line 373-375 of the the revised manuscript, we have replaced the“The results of Real-time PCR detection confirmed the localization and quantitative expression of LHR, which showed that from high to low of the expression of LHR mRNA i was LH group, control group and vitrification group (*P*<0.05) (Fig 6 D).” with “The findings from Western blot analysis corroborated the immunofluorescence and mRNA expression results (Fig 6 D and E)” and it was marked in red.

***6. Figure 7 D & E. From the data, the authors claim that the protein levels of Caspase are different between the control and LH groups, while mRNA doesn’t have differences. How is this accounted for? The authors state that active Caspases are in mRNA and protein. Please explain how the authors inferred this from a Real-time PCR. The protein is active.Authors should understand the expression levels of caspase are almost 3.5-fold. At the same time, Foxl2 and Lrg5 are marginal while making exuberant conclusions about the overexpression of Foxl2 and Lrg5 in the LH group compared to the control.***

**Response:**

We appreciate your valuable reminder to provide an explanation regarding the result that the protein levels of Caspase are different between the control and LH groups, while mRNA doesn’t have differences in Fig 7 D and E. We apologize for neglecting to discuss this result in the previous manuscript.

After repeating the experiment three times, we have included thorough discussions about these results in line 521-530 of our discussion in revised manuscript. Our explanations are primarily based on the the significantly high expression of active caspase-3 in the vitrification group has confirmed the effective effect of LH. Although there is no significant difference in mRNA expression between the LH group and the control group, it also proves that LH plays a positive protective role in the freeze-thaw process. This effect is as if the freeze-thaw process did not occur in the follicles and granulosa cells, that is, the protective effect of LH is of great significance. At the same time, there were differences in protein expression between the control group and LH group, but no differences in mRNA, which may have differences in protein and mRNA properties. The above discussion has been highlighted in red in the revised manuscript.

Since caspase-3 can only play its apoptotic role after activation, we directly selected the target gene as activated caspase-3 and directly anchored the activated caspase-3 through antigen and antibody binding to detect the expression of active caspase-3 in each group to avoid interference from silent caspase-3. Therefore, both protein and mRNA in our experiment were active caspase-3, rather than caspase-3.

***7.*** ***Figure 8. Results of functional in vitro studies involving ovarian explants are often contradictory. Several reports indicate an inhibitory effect of TGF-β1 on primary follicle survival and/or progression to the late preantral/early antral stage. However, other studies suggest positive effects or a lack of effect (Fortune 2003, Juengel & McNatty 2005). How do authors explain this?***

**Response:**

We greatly admire your insights and judgment on cutting-edge reporting in this field, and thank you for asking this valuable question that will take us further in this area of research. The question of the effect of TGF-β on follicles is also one that we are very concerned about and decided to verify.

Some studies believe that TGF-β has a positive effect on the development of follicles, but some studies still believe that TGF-β has an inhibitory effect on follicles or granulosa cells at different stages of development. In our experimental study conducted at the same time, the sequencing results of single cells that formed follicles in vitro showed significant high expression of TGF-β and its superfamily members. However, the specific effects and mechanisms of TGF-β on follicles at different developmental stages and granulosa cells from different sources need to be further verified. To this end, through a series of studies and verification, TGF-β and its super family members have a positive effect on follicles, but the specific process and mechanism of the role, as well as the differences in the role of follicles at different stages of development, need our further work to explore and prove.

The above discussion relates to the following references in this article:

[6]Zheng W, Zhang H, Liu K. The two classes of primordial follicles in the mouse ovary: their development, physiological functions and implications for future research. Mol Hum Reprod. 2014; 20: 286–292.

[40]Almeida CP, Ferreira MCF, Silveira CO, Campos JR, Borges IT, Baeta PG, et al. Clinical correlation of apoptosis in human granulosa cells-A review. Cell Biol Int. 2018; 42:1276-1281.

[41]Wei X, Zheng L, Tian Y, Wang H, Su Y, Feng G, et al. Tyrosine phosphatase SHP2 in ovarian granulosa cells balances follicular development by inhibiting PI3K/AKT signaling. J Mol Cell Biol. 2022; 14: mjac048.

[42]Peng M, Wang Y, Qiang L, Xu Y, Li C, Li T,et al. Interleukin-35 Inhibits TNF-α-Induced Osteoclastogenesis and Promotes Apoptosis via Shifting the Activation From TNF Receptor-Associated Death Domain (TRADD)-TRAF2 to TRADD-Fas-Associated Death Domain by JAK1/STAT1. Front Immunol. 2018; 9:1417.

[43]Zhao X, Li H, Chen X, Wu Y, Wang L, Li J. Long non-coding RNA MSTRG.5970.28 regulates proliferation and apoptosis of goose follicle granulosa cells via the miR-133a-3p/ANOS1 pathway. Poult Sci. 2023; 102:102451.

[46]Hobeika E, Armouti M, Kala H, Fierro MA, Winston NJ, Scoccia B, et al. Oocyte-Secreted Factors Synergize With FSH to Promote Aromatase Expression in Primary Human Cumulus Cells. J Clin Endocrinol Metab. 2019; 104:1667-1676.

***8.*** ***Discussion: Many results and conclusions were made from counting and tabulating cells; unfortunately, those are unavailable for review. The tables and explanations of the above comments are required to decide on this manuscript.***

**Response:**

We agree with your objective, scientific comments, and in order to make the results more reproducible and scientific, we have uploaded the laboratory protocol to protocols.io as requested by the journal, along with placing the DOI link in the methods section of the revised manuscript. At the same time, the original data of all blot images and "minimal data set" were uploaded by supporting information.
