# Supplementary material for: Protective effect of luteinizing hormone on frozen-thawed ovarian follicles and granulosa cells
Source: PLoS One. 2025 Jan 14;20(1):e0317416. doi: 10.1371/journal.pone.0317416 (PMC11731763; doi:10.1371/journal.pone.0317416)
Supplement: Attachment — Submitted filename: Response to Reviewers.docx [file pone.0317416.s004.docx]

**Responses to the** **Journal’ Comments**

***1. Please review your reference list to ensure that it is complete and correct. If you have cited papers that have been retracted, please include the rationale for doing so in the manuscript text, or remove these references and replace them with relevant current references. Any changes to the reference list should be mentioned in the rebuttal letter that accompanies your revised manuscript. If you need to cite a retracted article, indicate the article’s retracted status in the References list and also include a citation and full reference for the retraction notice.***

**Response:**

Thank you for your patient guidance on how to check the list of references. According to the journal requirements, we checked all the references article by article, and we confirmed that the references were complete and correct. At the same time, none of the 52 references we cited have been retracted. Among the 52 references, 4 have been added according to the comments of the reviewers. For this reason, starting from the 31^st^ reference, the serial number of the references has been modified accordingly and marked in red.

**Responses to the Reviewers’ Comments**

**Responses to the Comments of Reviewer 1:**

***1. As the Fig 2 indicate LH is having protective effect only in the TV group w.r.t control whereas morphology differs and cell viability decreases w.r.t control in both LH-AV and LH BV groups, the authors can discuss whether it is not the effect of LH in general but only when LH is present throughout (TV) that makes that difference.***

**Response:**

As for the supplementary discussion: "The intervention effect of LH in the whole process of vitrification and cryopreservation is significantly better than that of LH before or after vitrification cryopreservation", we thank you for your valuable reminder. We apologize for neglecting to discuss this result in the previous manuscript.

In our revised manuscript, we have included thorough discussions about these results in depth in the third paragraph of our discussion. Our explanations are primarily based on the protective effect of hormone regulation on follicles and the correlation between granulosa cells and follicle development. Detailed discussions have been added on pages 22-23, lines 432-435, 441-443, 453-455, and marked in red. We have taken precautions to ensure that the revised explanations are scientifically sound and well-supported by the available data.

Our revisions are quoted below for your convenience:

To directly observe the impact of vitrification and cryopreservation on granulosa cells and follicular structure, all morphological assessments were conducted on cryopreserved ovarian tissue. Histological analyses using HE staining demonstrated that continuous administration of LH during vitrification and cryopreservation significantly reduced nuclear shrinkage in oocytes and apoptosis in granulosa cells, typically caused by freeze-induced damage. This method provided enhanced protection to follicular morphology and structural integrity compared to interventions applied only before or after cryopreservation. Endocrine function plays an important role in ovarian growth and development. Therefore, the protective regulation of hormones in the process of ovarian tissue cryopreservation is the basic guarantee for the recovery of ovarian function [31,32]. The findings suggest that sustained LH exposure throughout the freeze-thaw cycle promotes granulosa cell proliferation and development during pre-culture and pre-equilibration phases, protects against cryodamage during vitrification, and facilitates cellular recovery during gradient thawing and subsequent culture. However, LH administration during the pre-culture and pre-equilibration phases alone offers protection only prior to freezing, failing to prevent damage during the freezing process itself. Studies have shown that the unprotected vitrification process can significantly damage the mitochondrial integrity of the granulosa cell layer and reduce the efficiency of follicle cryopreservation [33,34]. The granulosa cells across four groups were evaluated for viability using the LIVE/DEAD assay. The proportions of live cells in both the control and LH groups were significantly higher compared to the LH-BV and LH-AV groups, under the condition of equal total cell numbers per group. This suggests that LH supplementation during the freeze-thaw process offers the most effective protection. In the assessment of viable follicles at various developmental stages, groups receiving LH treatment exhibited a significantly greater number of secondary and mature follicles than the control group. This finding aligns with the hypothesis that LHR concentration is closely associated with follicular maturity and that LHR sensitivity to LH increases with follicular volume [35]. These results reinforce earlier findings. Therefore, we believe that only LH intervention in the whole vitrification cryopreservation process is the best intervention mode to improve the efficiency of follicle cryopreservation.

The references 31, 32 and 33, 34 in above paragraph are newly cited and marked in red. The new references are as follows:

[31]Wang F, Tian Y, Huang L, Qin T, Ma W, Pei C, et al. Roles of follicle stimulating hormone and sphingosine 1-phosphate co-administered in the process in mouse ovarian vitrification and transplantation. J Ovarian Res. 2023 Aug 24;16(1):173. doi: 10.1186/s13048-023-01206-1.

[32]Bromer JG, Patrizio P. Fertility preservation: the rationale for cryopreservation of the whole ovary. Semin Reprod Med. 2009 Nov;27(6):465-71. doi: 10.1055/s-0029-1241056.

[33]Fransolet M, Noël L, Henry L, Labied S, Blacher S, Nisolle M, et al. Evaluation of Z-VAD-FMK as an anti-apoptotic drug to prevent granulosa cell apoptosis and follicular death after human ovarian tissue transplantation. J Assist Reprod Genet. 2019 Feb;36(2):349-359. doi: 10.1007/s10815-018-1353-8.

[34]Godoy LC, Streit DP Jr, Zampolla T, Bos-Mikich A, Zhang T. A study on the vitrification of stage III zebrafish (Danio rerio) ovarian follicles. Cryobiology. 2013 Dec;67(3):347-354. doi: 10.1016/j.cryobiol.2013.10.002.Because of the insertion of new references in the discussion section, we have made corresponding changes to the serial number of references appearing in the paper, and marked them in red.

**Responses to the Comments of Reviewer 2:**

***1.*** ***“1ul of cDNA”in 2.14in the original manuscript has been deleted.And“3 μL RNA used for reverse transcription”has been added in 2.13 in the revised manuscript line 261. Please change it to amount not the volume used.***

**Response:**

Thank you very much for your careful review on our manuscript. At your suggestion, we have changed "3 μL of RNA" in line 261 of manuscript 2.13 to "1 μg of RNA " and marked in red.
